# Identification and validation of a prognostic model for melanoma patients with 9 ferroptosis-related gene signature

**DOI:** 10.1186/s12864-022-08475-y

**Published:** 2022-03-30

**Authors:** Yuxuan Chen, Linlin Guo, Zijie Zhou, Ran An, Jiecong Wang

**Affiliations:** 1 Department of Plastic Surgery, Union Hospital, Tongji Medical College, Huazhong University of Science and Technology, Wuhan, PR China; 2 Department of Neurosurgery, Union Hospital, Tongji Medical College, Huazhong University of Science and Technology, Wuhan, PR China

**Keywords:** Melanoma, Ferroptosis, Prognosis

## Abstract

**Background:**

Melanoma is a highly heterogeneous and aggressive cutaneous malignancy. Ferroptosis, a new pathway of cell death depending on the intracellar iron, has been shown to be significantly associated with apoptosis of a number of tumors, including melanoma. Nevertheless, the relationship between ferroptosis-related genes (FRGs) and the melanoma patients’ prognosis needs to be explored.

**Methods:**

Download expression profiles of FRGs and clinical data from The Cancer Genome Atlas (TCGA) database. 70% data were randomly selected from the TCGA database and utilized the univariate Cox analysis and the least absolute shrinkage and selection operator (LASSO) regression model to create a prognostic model, and the remaining 30% was used to validate the predictive power of the model. In addition, GSE65904 and GSE22153 date sets as the verification cohort to testify the predictive ability of the signature.

**Results:**

We identified nine FRGs relating with melanoma patients’ overall survival (OS) and established a prognostic model based on their expression. During the research, patients were divided into group of high-risk and low-risk according to the results of LASSO regression analysis. Survival time was significantly longer in the low-risk group than that of in the high-risk group (*P* < 0.001). Enrichment analysis of different risk groups demonstrated that the reasons for the difference were related to immune-related pathways, and the degree of immune cell infiltration in the low-risk group was significantly higher than that in the high-risk group.

**Conclusions:**

The FRG prognostic model we established can predict the prognosis of melanoma patients and may further guide subsequent treatment.

**Supplementary Information:**

The online version contains supplementary material available at 10.1186/s12864-022-08475-y.

## Introduction

Melanoma is a malignant tumor around the world which is associate with rapid growth, early metastasis, local recurrence and poor prognosis [1]. According to the data of Cancer statistics in 2020, the incidence of melanoma accounted for 7% and 4% of male and female patients respectively, ranking fifth and sixth respectively [2]. Worldwide, there are about 23,100 new reported cases of melanoma every year and about 55,500 deaths due to melanoma ranked as the sixth most common malignancy in the US. Therefore, early detection and recognition of melanoma are keys to improve survival rate. At present, the treatment of the skin melanoma mainly includes surgical resection, which is the standard therapy for the primary melanoma, and other treatments applied to treat advanced melanoma such as radiotherapy, chemotherapy and immunotherapy [3]. Although thorough surgical resection of the tumor can greatly improve the five-year survival rate of patients with melanoma [4], the prognosis of patients are still not satisfactory, with a 5 year survival rate of 40 − 50% especially for the lymphatic metastasis and gene mutations [4]. Specific biomarkers play an important role in the early screening, diagnosis, and prognosis of melanoma. Therefore, it is urgent to establish a more sensitive prognostic models to assess the patient’s current condition for monitoring recurrence and evaluating prognosis.

Ferroptosis is a non-apoptotic form of cell death dependent on intracellular iron, which is different from apoptosis, necrosis and autophagy [5], The occurrence of ferroptosis is closely related to the inhibition of glutathione peroxidase4 (GPX4) synthesis. The weakening of GPX4-dependent antioxidant defense system eventually leads to the accumulation of lipid ROS which is toxic to cells and the depletion of polyunsaturated fatty acids, finally resulting in cell death [6]. Besides, ferroptosis has been proved to be related to the prognosis of tumor [7]. Ubellacker et al. found that melanoma cells in lymph nodes may be swollen through incorporate oleic acid and other antioxidants to protect themselves from ferroptosis [8]. This may be one of the reasons for the earlier lymphatic metastasis of melanoma mentioned above. Therefore, it can be speculated that the relationship between the expression of FRGs and the prognosis of patients remains to be further explored and studied.

By downloading the mRNA expression profiles of some melanoma patients from the Cancer Genome Atlas (TCGA) and their corresponding clinical data, and combining with the FRGs obtained from the original pubmed literatures, we constructed a prognosis model related to ferroptosis gene expression, and used the mRNA expression profiles of the remaining melanoma patients in the TCGA database combined with their clinical data to verify the correctness of the model. Finally, the functional enrichment and tumor microenvironment were analyzed to explore the possible mechanism.

## Materials and methods

**Fig. 1:**
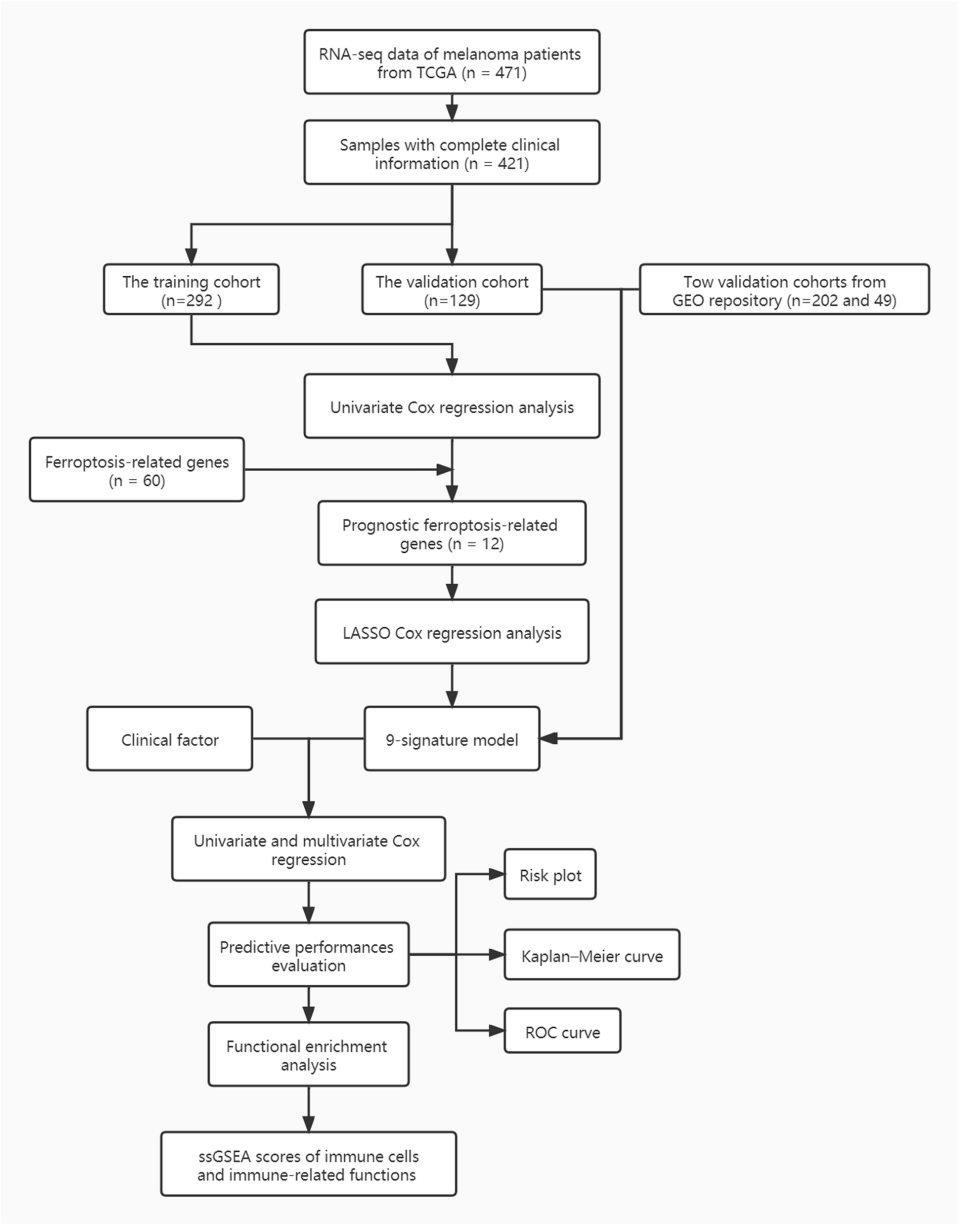
The procession flow diagram in the present study

The research flowchart is displayed in Fig. 1.

### Data Collection and Preprocessing

First of all, we downloaded the mRNA expression profiles and corresponding clinical information of 471 melanoma patients from the TCGA database (https://portal.gdc.cancer.gov/), after applying the “scale” function in the “limma” R package (version 4.0.3) to normalize data among databases, we combined them together. After that, removing incomplete clinical data and 0-days follow-up duration from samples, 455 melanoma samples obtained and used for the primary cohort. By using the method of generating random numbers by Microsoft Office Excel (version 2019), 455 samples are randomly divided into parts accounting for 70% of the total number of samples (*n* = 318) to be the training cohort and 30% of the total number of samples (*n *= 137) to be the validation cohort. Table S1 lists the clinical characteristics of the patients above. After browsing the previous literatures [9–12], 60 FRGs were obtained. These genes will be shown in Supplementary Table S2.

### Identification of FRGs Affect Prognosis

After associating the expression levels of FRGs and melanoma patients’ overall OS in the training cohort, and trough univariate Cox analysis, 12 genes with prognostic significance (*p* < 0.05) were regarded as serving as an independent predictor for OS were obtained. These genes were used to form a prognostic model.

### Construction and Validation of the Prognostic Model

Through the least absolute shrinkage and selection operator (LASSO) Cox regression analysis with package “glmnet” in R software and associated 12 genes above and patients’ survival data to find the most significant 9 genes and their corresponding Cox regression coefficient to build model. This model offered a formula to calculate risk score of each patient: risk score=e^sum (each gene’s expression×corresponding coefficient)^. According to the median value of the risk score in the training cohort with R software, patients in the training cohort were divided into high-risk group and low-risk group. After getting the formula obtained by establishing the model through training cohort, using this formula to calculate each patient’s risk score in validation cohort from TCGA database and divide them into high and low risk groups for the validation of the model. The t-distributed stochastic neighbor embedding (t-SNE) and principal component analysis (PCA) was analyzed with “Rtsne” package and the prcomp function in the “stats” package to explore the distribution of high and low-risk groups. By comping the survival between the two groups above and evaluate the model’s predictive ability using the “survivalROC” package in R respectively with “timeROC” package, Kaplan–Meier survival curves and a time-dependent receiver operating characteristic curve (ROC) curve analysis were employed. Besides, we downloaded the GSE65904 and GSE22153 datasets to verify the predictive power of the model, the two datasets are embedded in GPL10558 (Illumina HumanHT-12 V4.0 Expression BeadChip) and GPL6102 (Illumina Human-6 V2.0 Expression Beadchip) platforms, respectively. After downloading the Series Matrix file of the data set, the clinical information of patients was extracted, and the probes were replaced with the gene ID by the annotation information of the corresponding platform. The samples with incomplete clinical information were eliminated after the matrix of the combination of patient gene expression profile and clinical information, and two datasets with 202 and 49 patient samples were obtained, respectively. After using the RemoveBatchEffect function in “limma” R package (Version 4.0.3) to remove the batch effect between TCGA data set and Gene Expression Omnibus (GEO) repository (https://www.ncbi.nlm.nih.gov/geo), the “scale” function in “limma” R Package (Version 4.0.3) was used to normalize the data. Finally, calculating patient risk scores by the formula obtained in the train cohort and applying survival analysis and analysis of AUC in ROC for risk signature at 1-, 2- and 3- year survival time in the same way mentioned above.

### Functional analysis

With the “clusterProfiler” R package, the analysis of Kyoto Encyclopedia of Genes, Gene Ontology (GO) and kyoto encyclopedia of genes and genomes (KEGG; www.kegg.jp/kegg/kegg1.html ) between the groups of high- and low-risk was enriched. In order to investigate the pathways enriched in the subgroups of high- and low-risk and explore possible molecular mechanisms. Using the false discovery rate (FDR) method to adjust *P*-values and while the *P*-value < 0.05, the pathways were considered to be enriched significantly.

### Immune score, calculation of immune-related pathways and immune cells infiltration between two groups

By using the “estimate” package of R software in order to estimate the expression of immune and stromal cells in malignant tumor tissue and obtain immune stromal component ratio in the tumor microenvironment (TME) [13]. The result of the “estimate” R package created three scores to evaluate the presence of stroma (Stromal Score), the level of immune cells infiltrations (Immune Score), and the sum of stromal score and immune score (Estimate Score) [14]. The scores go up with the proportion of corresponding condition in the TME. The single-sample geneset enrichment analysis (ssGSEA) [15] was finished by using “GSVA” package of R software, the result of the level of the 13 immune-related pathways expression and16 kinds of immune-related cells are received. Supplementary Table S3 illustrates the immune-related genes.

### Statistical analysis

Multivariate and Univariate Cox regression analyses were used to estimate if the factor can be regarded as an independent predictor. Utilizing R package “timeROC” to predict overall survival. Using t-test and chi-square test of student to identify the difference of Stromal Score, Immune Score, and ESTIMATE Score between patients in different risk groups. R software (Version 4.05) were applied here for all statistical analyses. Statistically significant were regarded in case when p-value less than 0.05.

### Quantitative real-time PCR

Human normal skin cell line (TE353.sk) and human melanoma cell line (SKMEL5) (purchased from YaJiBiological, China) for verification are prepared after culture in accordance with the instructions provided by the manufacturer. Before the 2 ^−ΔΔCt^ statistics were applied to calculate the gene expression level in the final step, the total RNA of two kinds of cells above was extracted by TRIzol reagent (Invitrogen, China), and PrimeScript RT kit (Takara, China) was used for reverse transcription and the SYBR PrimeScript RT-PCR kit (Takara, China) was used for quantitative reverse transcriptase polymerase chain reaction (qRT-PCR) analysis. Supplementary Table S4 includes the primer sequences involved in this study.

## Results

### Identification of prognostic FRGs

**Fig. 2:**
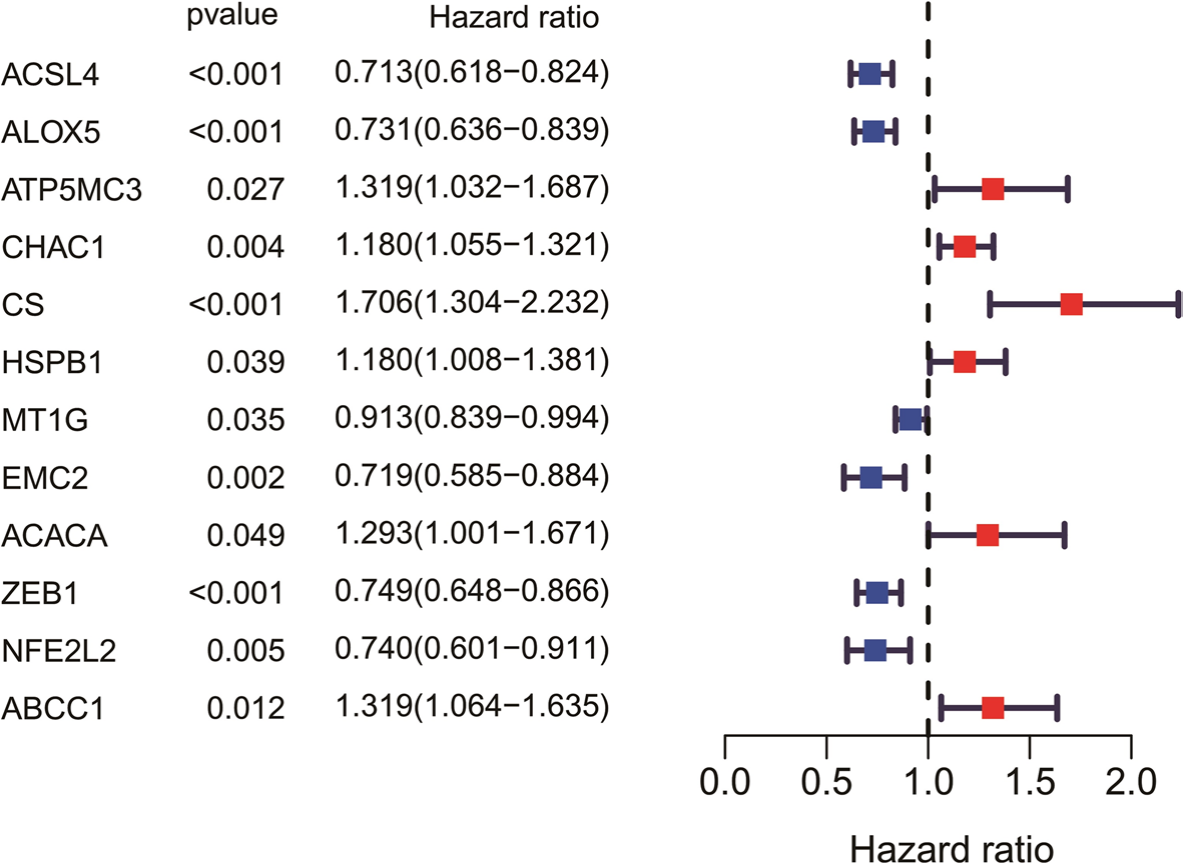
Forest plots to demonstrate the univariate Cox regression analysis results between gene expression and the OS of melanoma patients

Univariate Cox regression analysis showed that 12 genes could be used as independent factors of OS in patients with melanoma. Among the 12 genes, 6 of them were protective genes (HR<1) and 6 were risk genes (HR > 1) (Fig. 2).

### Prognostic model construction in the training cohort

**Fig. 3:**
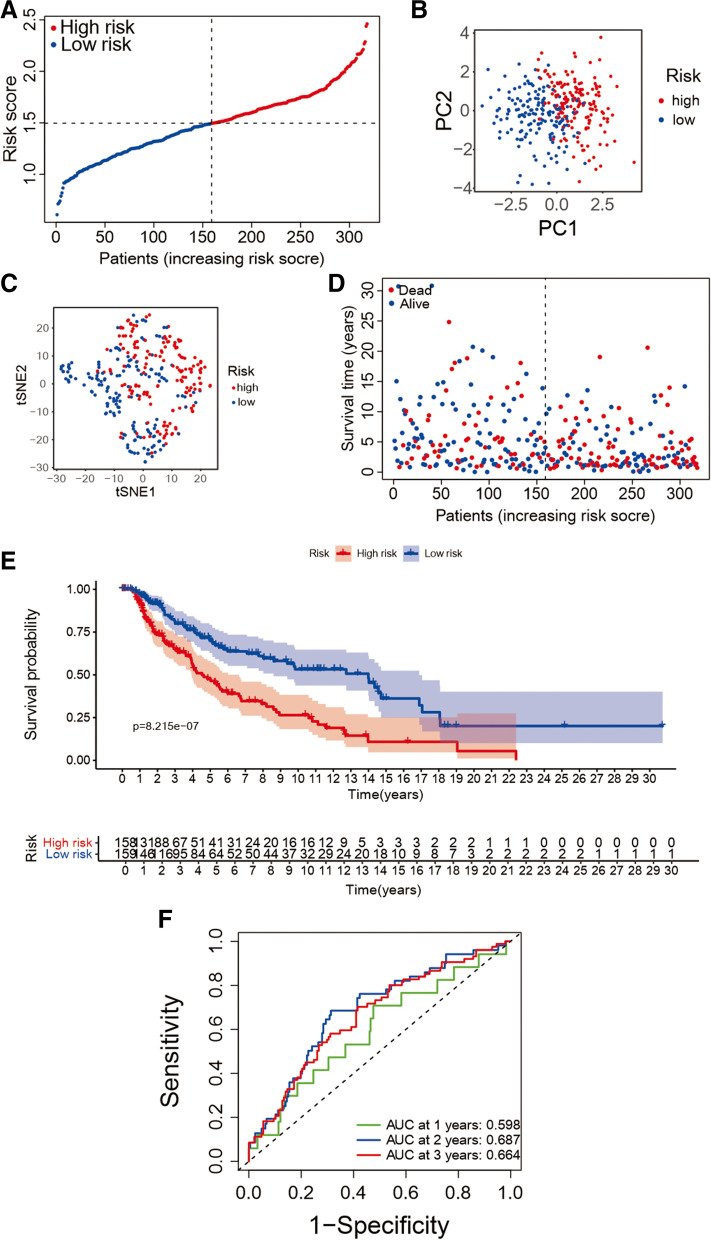
The 9-gene model in the train cohort prognostic analysis. **A** The risk score distribution in the train cohort. **B** the train cohort PCA analysis. **C** t-SNE plot the train cohort. **D** The distribution of OS in the train cohort. **E** The Kaplan–Meier survival OS analysis between the high-risk and low-risk group of the train cohort. **F** The analysis of AUC in ROC for risk signature at 1-, 2- and 3-year survival time in the train cohort

Nine genes which are significant subject to the OS of melanoma patients were obtained after the utilizing of LASSO Cox regression analysis (i.e., ACSL4, ALOX5, ATP5MC3, CHAC1, CS, MT1G, ACACA, ZEB1, and ABCC1). A ferroptosis-related prognostic model was created on account of the best value of λ from LASSO Cox regression analysis. The score of risk can be figured out though the following formula: risk score=e (0.05785 * expression level of ACACA − 0.14149 * expression level of ACSL4 − 0.06033 * expression level of ALOX5 + 0.04117 * expression level of ATP5MC3 + 0.02936 * expression level of CHAC1 + 0.25160 * expression level of CS − 0.00617 * expression level of MT1G − 0.09239 * expression level of ZEB1 + 0.18878 * expression level of ABCC1). The risk values of patients in the train cohort were arranged from high to low, and the median value was found as the cut-off value to divide patients into two groups of low-risk (*n* = 159) and high -risk (*n* = 159) (Fig. 3(A)). PCA and t-SNE analysis showed the result that the patients in low-risk and high-risk groups were split into two directions (Fig. 3(B) and (C)). Figure 3(D) indicated that patients of high-risk group own a poor survival. The Kaplan–Meier survival analysis also confirmed the survival time of high-risk group was yielding reduced (Fig. 3(E), *P* < 0.001). An evaluation of the predictive performance was evaluated with the time-dependent ROC curves of the model and in Fig. 3(F), the area under curve (AUC) reached 0.598 at 1 year, 0.687 at 2 years, and 0.664 at 3 years.

### The prognostic model validation in the validation cohort

**Fig. 4:**
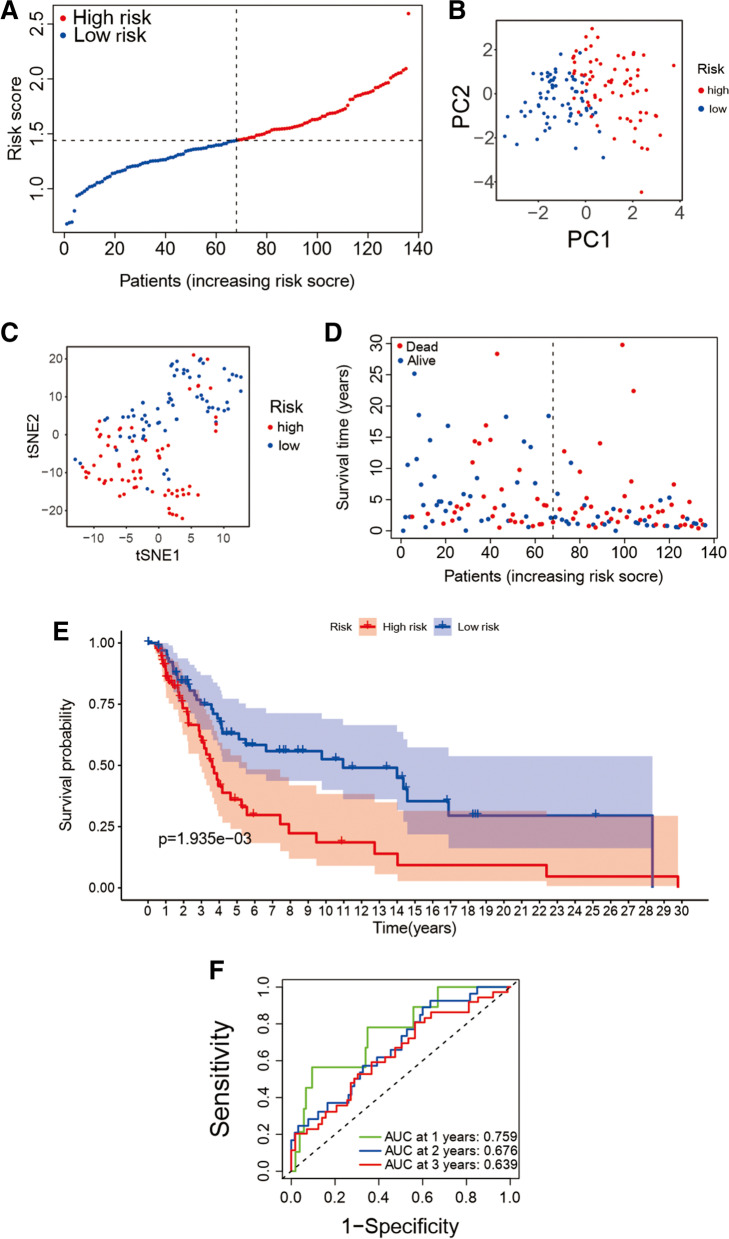
The prognostic model validation in the validation cohort. **A** The risk score distribution of in the validation cohort. **B** the validation cohort PCA analysis. **C** the validation cohort T-SNE plot. **D** The OS distribution in the validation cohort. **E** The Kaplan–Meier survival OS analysis between the high-risk group and low-risk group in the validation cohort. **F** The risk signature AUC in ROC analysis for 1-, 2- and 3-year survival time in the validation cohort

Calculating the score of risk for each patient in the validation cohort by using the formula obtained above to verify the accuracy of the prediction ability of the model. Using the method mentioned above, patients are split into high- (*n* = 68) and low-risk (*n* = 68) groups in the validation cohort according to risk score and median (Fig. 4(A)). In the validation cohort, a reliable clustering ability of risk score are also validated by PCA and t-SNE analysis (see Fig. 4(B) and 4(C)). It is obvious that the survival time of high-risk group patients is shorter than that of low-risk (Fig. 4(D), *P* < 0.001). Meanwhile, the Kaplan–Meier survival OS analysis between the high-risk group and low-risk group in the validation cohort shows there were also significant differences in survival between high and low-risk groups. (Figure 4(E)) and the values of AUC of year 1, 2 and 3 after ROC analysis are 0.759, 0.676 and 0.639 (Fig. 4(F)).

### Independent prognostic value of the risk score

**Fig. 5:**
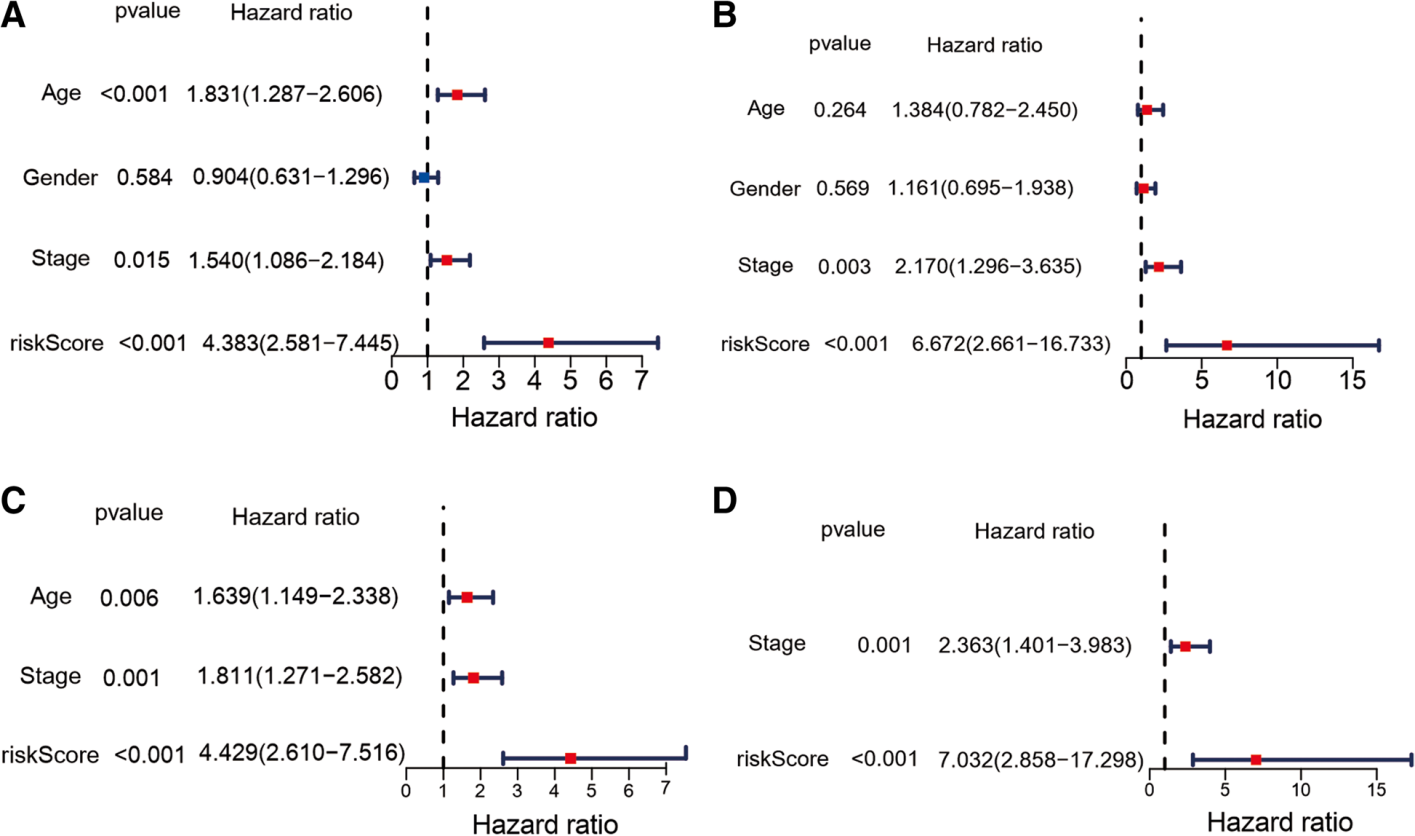
Independent prognostic signature presented by risk score for melanoma. The univariate and multivariate Cox regression OS analyses results in the validation cohort (B/D) and in the train cohort (A/C)

In order to further explore whether risk score is an independent prognostic factor, we conducted univariate and multivariate Cox regression analysis. In the train cohort (HR= 4.383, 95% CI= 2.581 − 7.445, *P* < 0.001, Fig. 5(A)) and the validation cohort (HR= 6.672, 95% CI= 2.661 − 16.733, *P* < 0.001, Fig. 5(B)), univariate Cox regression analysis showed that the both risk score had a significant relationship with OS. Multivariate Cox regression analysis corrected for potential confounding factors also showed that risk score could be used as an independent predictor of OS (train cohort: HR= 4.429, 95% CI= 2.610 − 7.516, *P* < 0.001; validation cohort: HR= 7.032, 95% CI= 2.858 − 17.298, *P* < 0.001, Fig. 5(C) and 5(D)). In order to prove that our prognostic model is not a different way to quantify immune infiltration, we include the ESTIMATE score into the multivariate regression analysis model. The results show that the FRG based risk score still has predictive ability (*P* < 0.001 in train cohort and *P* < 0.05 in test cohort, Figure S1A and Figure S1B) although with a diminished hazard ratio.

### Functional analyses

**Fig. 6:**
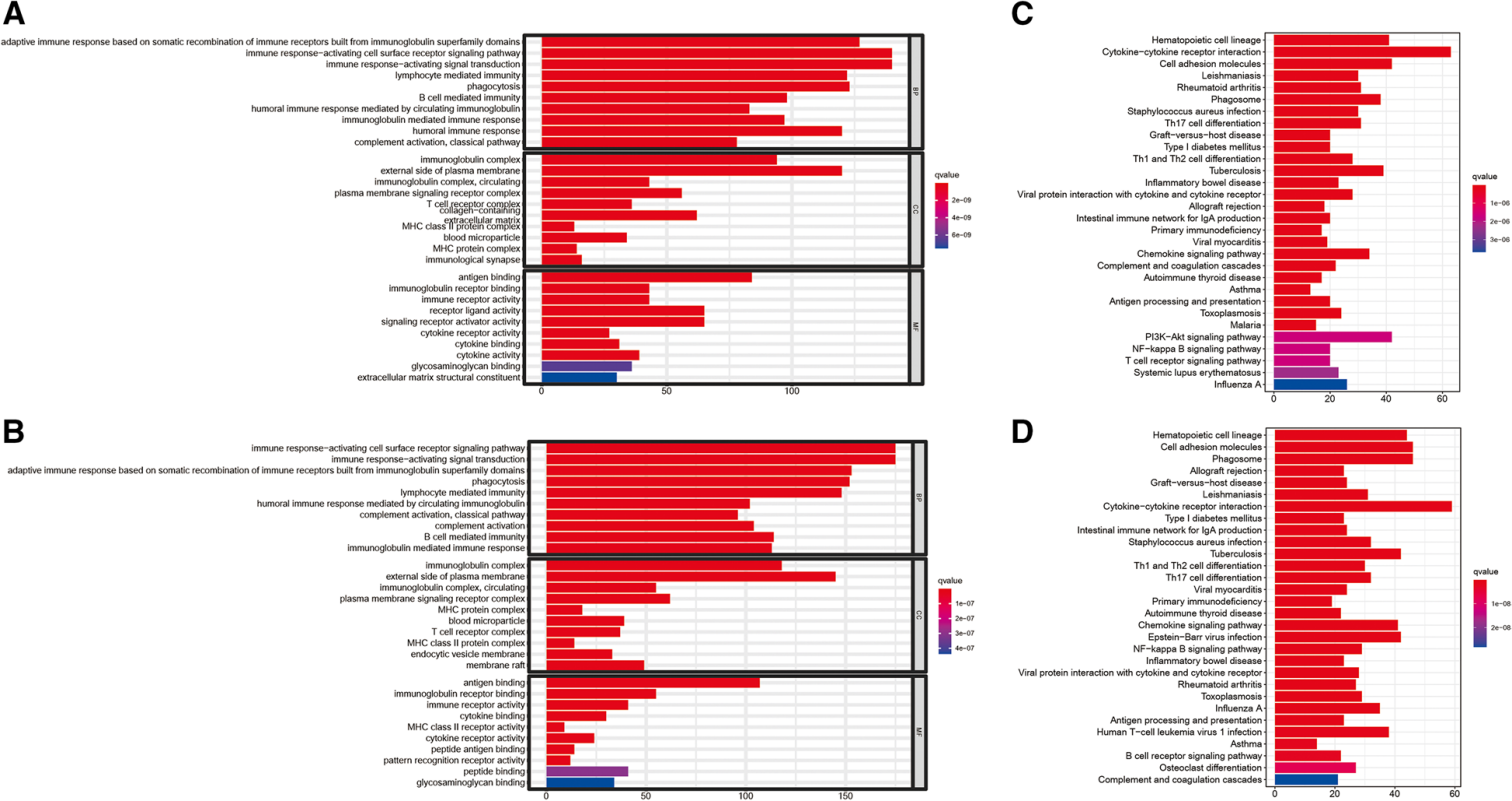
The results of functional analyses. The barplot graph for the result of GO enrichment analysis for the train cohort (**A**) and the validation cohort (**B**). The barplot graph for the result of KEGG pathway analysis for the train cohort (**C**) and the validation cohort (**D**)

Enrichment and KEGG pathway were carried out according to the differential expression genes (DEGs) between high-risk group and low-risk group in the train cohort and validation cohort to clarify the biological function and pathway related to risk score. According to the GO enrichment analysis result, the DEGs between risk groups from validation cohort and the train cohort were primarily enriched in immune response−activating cell surface receptor so as to signal pathway and immune response−activating signal transduction. (*P*. adjust <0.05, Fig. 6(A), (B)). It is can be seen that the cytokine−cytokine receptor interaction pathway was significantly enriched in both cohorts (*P*. adjust <0.05, Fig. 6(C), (D)) in KEGG pathway analysis.

### The Immune-related pathways and immune cells infiltration analysis

**Fig. 7:**
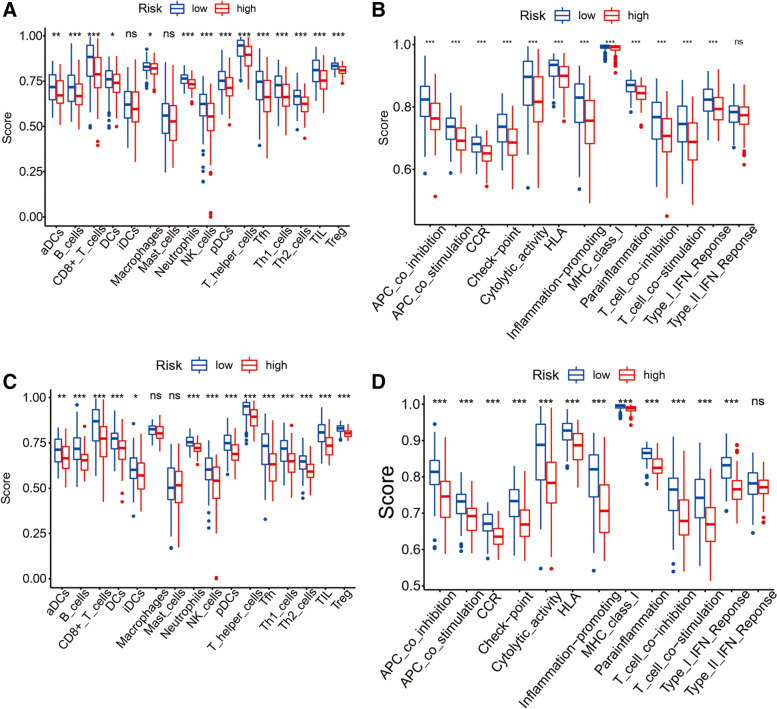
Differences of immune-related pathways and immune cells infiltration between groups of high- risk and low- risk. ssGSEA scores of 16 immune cells (**A**) and ssGSEA scores of 13 immune-related functions (**B**) in the train group. ssGSEA scores of 16 immune cells (**C**) and ssGSEA scores of 13 immune-related functions (**D**) in the validation group. ns: not significant; ∗: *P* < 0.05;∗∗: *P* < 0.01; ∗∗∗: *P* < 0.001

In order to explore the difference of immune-related function between risk groups of high and low, each patient was scored by ssGSEA analysis, and then analyzed the enrichment of immune-related pathways and immune cells by the score of each patient. In the train cohort, B cells, D8 + T cells, NK cells, Neutrophils, pDCs, T helper cells, Th1 cells, Th2 cells, Tfh, TIL, T reg along with other immune cell subsets between the two groups were significantly different and down-regulated in the immune-related pathway, in the high-risk group (adjusted *P* < 0.05, see Fig. 7 (A)), Cytolytic activity, Inflammation-promoting was significantly down-regulated In the validation cohort, in the high-risk group (adjusted *P* < 0.05, Fig. 7 (B). the enrichment of Macrophage, Dendritic Cells (DCs) and Mast cells was different from that of train group, and the enrichment of remaining immune cells and the pathways of immune-related function was similar to that of train cohort (corrected *P* < 0.05, Fig. 7 (C) and (D)).

### Verification of risk score model in GEO repository

**Fig. 8:**
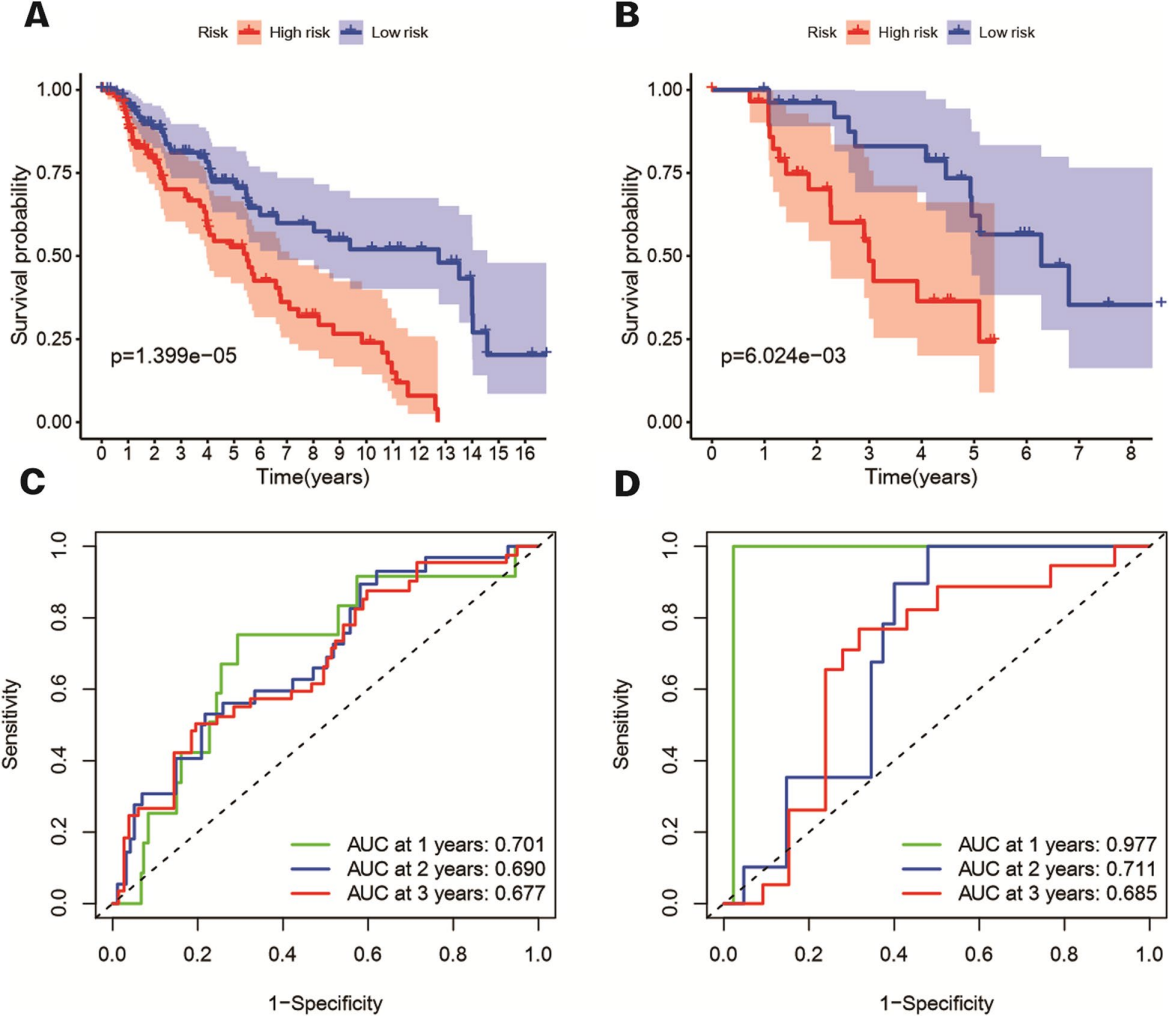
The Kaplan–Meier survival OS analysis between the high-risk and low-risk group of the validation cohort 1 (**A**) and validation cohort 2 (**B**). The analysis of AUC in ROC for risk signature at 1-, 2- and 3-year survival time in the validation cohort 1 (**C**) and validation cohort 2 (**D**)

Data from GSE65904 and GSE22153 are used as validation cohorts 1 and validation cohorts 2. By using our FRG based riskScore, the samples of the two verification cohorts are divided into high and low risk groups. The Kaplan–Meier survival analysis shows the difference in survival time between low and high-risk groups still significant in two verification cohorts. (Figure 8(A), *P* < 0.001 and (B), *P* < 0.05). The result of time-dependent ROC curves was display in Fig. 8(C/D) to evaluate the predictive performance of the model. The area under curve (AUC) reached 0.701 at 1 year, 0.690 at 2 years, and 0.677 at 3 years for validation cohorts 1 and 0.977 at 1 year, 0.711 at 2 years, and 0.685 at 3 years for validation cohorts 2.

### Genes expression levels in cell lines validation

**Fig. 9:**
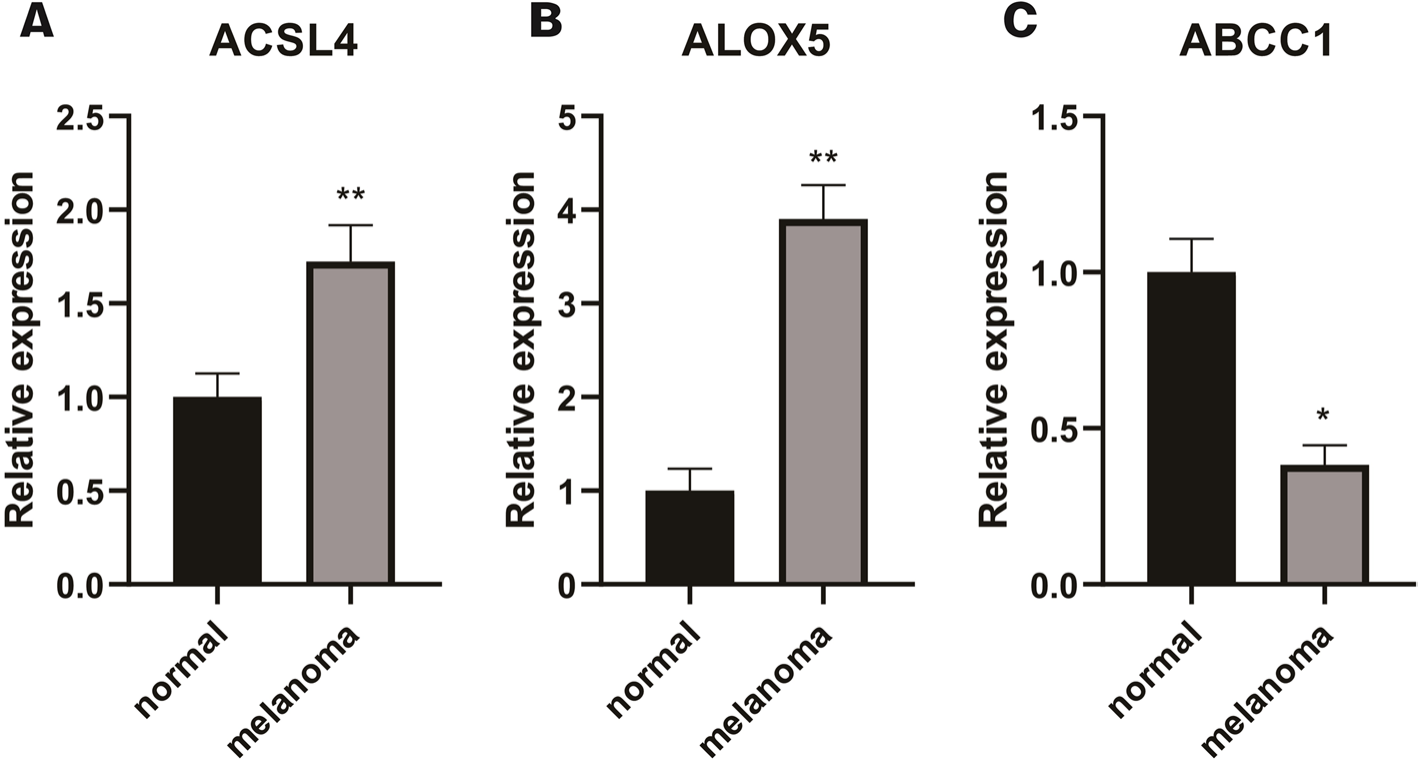
Differences of Genes Expression Levels in Normal Skin and melanoma Cell Lines Validated by qRT-PCR. Relative expression levels of **A** ACSL4, **B** ALOX5, and **C** ABCC1 between normal skin cell line and melanoma cell line. ns: not significant; ∗*P *< 0.05; ∗∗*P *< 0.01; ∗∗∗*P *< 0.001; ∗∗∗∗*P *< 0.0001

In the 9 gene signatures, by qRT-PCR, we found that significant differences in the expression of ABCC1, ACSL4 and ALOX5 between normal skin cell lines and melanoma cell lines. Among them, the expression of ACSL4 and ALOX5 was up-regulated in normal skin cell lines (Fig. 9 (A) and (B), *P* < 0.05), along with the higher expression level of ABCC1 in melanocyte lines (Fig. 8 (C), *P* < 0.05).

## Discussion

In this study, we used the patients whose mRNA expression profiles and clinical data of 70% melanoma in the TCGA database as the train cohort and combined with 60 FRGs to construct a model containing 9 FRGs to predict the prognosis of patients of melanoma. The remaining 30% patients, data were used as the verification group to verify the model predictive function.

In the above two cohorts, the patients’ survival time in the high-risk group was significantly shorter than that in the group of low-risk. Analysis with Functional enrichment showed that the differences were mainly because of cytokine-cytokine receptor interaction and immune response-related pathways. Besides, immune-related functions and immune cell infiltration in the group of high-risk were remarkably lower than those in the group of low-risk. A signature model consisting of 9 ferroptosis genes, acyl-CoA synthetase long-chain family member 4(ACSL4), 5-Lipoxygenase (ALOX5), metallothionein (MT)-1G and Zinc finger E-box-binding homeobox 1 (ZEB1) as protective genes, ATP synthase membrane subunit c locus 3 (ATP5MC3), ChaC glutathione specific gamma-glutamylcyclotransferase 1 (CHAC1), citrate synthase (CS), acetyl-CoA carboxylase alpha (ACACA), ATP binding cassette subfamily C member 1 (ABCC1) as risk genes. Ferroptosis is a regulatory necrotic cell death controlled by glutathione peroxidase 4(GPX4), and the overexpression of ACSL4 will reduce the expression of GPX4. Moreover, ACSL4, a member of the long-chain acyl-CoA synthase family, can induce ferroptosis by oxidizing arachidonic acid [16, 17]. Therefore, ACSL4, a proferroptotic gene, plays a crucial role in cells ferroptosis process. Sebastian Doll et al. found that ACSL4 knockout cells showed significant resistance to ferroptosis, and reexpression of ACSL4 enabled cells to regain sensitivity to ferroptosis [18]. Jing Cheng et al. observed that solafenib increased cell viability by reducing siRNA-mediated ACSL4 silencing, suggesting that ACSL4 may protect glioma cells and inhibit their proliferation via activating a ferroptosis pathway [16]. It can be concluded that the high expression level of ACSL4 can lead more tumor cells to ferroptosis. As shown in our prognostic model, the HR value of ACSL4 is less than 1, which means that it is a protective gene, the higher its expression level brings the better the patients’ possible prognosis. It is worth mentioning that in José Pedro et al. pointed out that ASCL4 may activate and attract immune cells to clear ferroptosis tumor cells by sending signals such as “find me” and “eat me” to immune cells by participating in ferroptosis tumor cells, and put forward the related conjecture that the loss of these signals may lead to immune evasion of tumor cells [19]. ALOX5 is an iron-containing non-heme dioxygenase, which seems a key enzyme in the synthesis of leukotriene and also can be used to catalyze the peroxidation of polyunsaturated fatty acids [20]. Ferroptosis could be irritated by lipid peroxidation to mediate inflammation-related cell death [21], and ALOX5 plays a crucial role in both wire death and inflammation [22]. Previous study found that inhibition of ALOX5 expression could decrease ferroptosis in nerve cells derived from hemorrhagic stroke mice [22]. Faronato et al. [23] and Miess et al. [24] thought that the expression of ALOX5 in clear cell renal cell carcinomas deficient in von Hippel-Lindau (VHL) gene was greatly increased, which may be that this kind of cancer cell needs more eicosanoids synthesized through ALOX5 expression to promote local inflammatory response. In the prognostic model of melanoma patients with FRGs created by Xu and Chen [25], ALOX5 also formed their 5 gene signature as an indispensable member, and it was confirmed that its expression can be used as an independent prognostic factor to predict the OS of patients. Metallothioneins (MTS) is a protein that is highly expressed under the influence of different environmental stressors and is closely related to heavy metal detoxifications and antioxidants. The up-regulation of MT-1G expression can increase the drug resistance of tumor cells to sorafenib by inhibiting ferroptosis [26]. However, Sun, X et al. found that sorafenib can activate Nrf2 through the cystathionase pathway leading to the expression of MT-1G in liver cells. Nevertheless, inhibiting the expression of MT-1G will enhance the metastatic tumor activity of sorafenib against hepatoma [27]. In conclusion, MT-1G may become a new target for anti-tumor therapy in the future. ZEB1 is known as an epithelial marker to down-regulate the expression of e-cadherin to affect the epithelial-mesenchymal transition and thus participate in the invasion and metastasis of tumor cells [28]. Lee and his team tested the results of ferroptosis inducers induction in HNC cell lines or EMT inhibition and rat neoplasm graft models, found that overexpression of ZEB1 may enhance the neoplasm cells sensitivity to ferroptosis, and it absolutely was additionally confirmed in animal models that the neoplasm volume within the overexpression group was considerably reduced compared with the control group [29]. ATP5G3 encodes a fractional monetary unit of mitochondrial membrane ATP synthase, that catalyzes ATP synthesis throughout organic process. ATP5G3 was found to be up-regulated three days when the prevalence of secondary craniocerebral injury to accelerate ferroptosis of cells [30]. CHAC1, a γ-glutamyl cyclotransferase, which inhibits ferroptosis by enhancing the degradation of glutathione [31, 32]. In the research and exploration of Wang et al., finally found that Artesunate may increase the expression of CHAC1 through the ATF4-CHOP pathway, thereby rising the sensitivity of Burkitt’s cancer cells to ferroptosis [33]. CS will catalyse the synthesis of change state from oxalacetate, And gives material for carboxylic acid synthesis thus on provide needed macromolecule precursors for macromolecule peroxidation caused by ferroptosis [34]. Erastin is one of the small molecules that can induce ferroptosis [35]. Dixon et al. found that silencing CS can significantly reduce ferroptosis induced by Erastin [5]. ACACA mainly acts on the first stage of fatty acid synthesis, and is one of the rate-limiting enzymes that regulate fat and metabolism, and plays a vital role in the tumor cells survival [36]. In addition, knockout of ACACA could inhibit drug-induced cell ferroptosis [37]. Meanwhile, AMPK pathway could be activated to inhibit its downstream ACACA, subsequently slowing down lipid accumulation and ferroptosis [38]. The expression of ABCC1 can be positively regulated by the antioxidant transcription factor Nrf2 [39] to regulate the process of cell ferroptosis. Cao et al. down-regulated the expression of ATP binding cassette (ABC)-family transporter multidrug resistance protein 1 (MRP1). This prevents glutathione from flowing out of the cells and effectively inhibits ferroptosis [40]. In melanoma, the synergistic effect of ABCC1 and glutathione S-transferase M1 can also make tumor cells resistant to vincristine [41].

GO and KEGG enrichment analysis showed that the difference in expression between groups was mainly related to tumor microenvironment, and the enrichment of signaling pathway of immune response-activating cell surface receptor, transduction of immune response-activating signal and cytokine-cytokine receptor interaction pathway were the most significant. We can find that cAMP signal pathway is highly enriched in these differentially expressed genes. Arumugham et al. found that cAMP can regulate T cell activation and immune synaptic assembly to regulate the immune process [42]. These three immune-related pathways have also been found to be enriched in lung adenocarcinoma [43], testicular cancer [44], glioblastoma [45], and other tumors and can be used to predict the prognosis of patients. Although the specific mechanism of their role in melanoma needs to be further studied, we think that they have great potential as indicators for predicting the prognosis of patients with melanoma. We found that there was a significant difference in immune cell infiltration between the group of high- and low-risk. The immune cell infiltration in the group of low-risk was higher than that in the group of high-risk, including CD8+T cell and DCs. DCs were found to accumulate large amounts of lipids and polyunsaturated fatty acids in tumor patients, which led to a decline in their ability to present antigens and unable to fully stimulate activated CD8+T cell [46, 47]. *In vitro* experiments conducted by Matsushita et al. also confirmed that CD8+T cells can increase the specific lipid peroxidation of ferroptosis by releasing interferon-γ and increase the occurrence of ferroptosis, thus improving the effect of immunotherapy [48]. This phenomenon may be due to the releasing signal molecules such as interferon-γ by DCs and CD8+T cells to activate ferroptosis [49]. The level of immune cell infiltration in the high-risk group is significantly lower than that in the low-risk group, indicating that the tumor cells in the high-risk group are less sensitive to ferroptosis. In accordance, Friedmann Angeli et al. also found that tumor cells with ferroptosis release arachidonic acid as an immune activator to obtain drug resistance and immune evasion from other tumor cells [19].

In this study, the risk value calculated after the establishment of the model is a reliable independent prognostic index. Compared with the conventional prognostic indexes such as “age”, “gender”, “tumor stage” and “ESTIMATE score”, the risk score created by the above nine gene expression can better predict the survival of patients, which also confirms that the gene-based expression signal can accurately predict the prognosis of patients with melanoma. However, this study also has some limitations. First of all, the clinical information in TCGA database is incomplete, especially the lack of treatment-related information. Secondly, the prognostic prediction model constructed in this study is based on retrospective data, and no prospective clinical studies have been carried out to verify the model. Garg et al. proposed a prognostic signature consisting of 121 metastasis-related genes to predict the prognosis of patients with melanoma [50]. But unfortunately, none of them appeared in both their and our signatures. Ubellacker et al. found that a high level of ferroptosis inhibitor glutathione peroxidase 4 (GPX4) may be the reason for the earlier lymphatic metastasis of melanoma, but interestingly it appears also not in the metastasis-related gene signature constructed by Garg et al. Whether the genes or ferroptosis-related genes in our signature are related to the risk of metastasis is the focus of our next work and research. Since our prognostic model is based on ferroptosis-related genes, as mentioned in our Discussion section above, the ferroptosis behavior of tumors is closely related to immune infiltration and immune pathway, there may be some overlap between ESTIMATE score and riskScores to evaluate immunity. More specific forms of interaction between the two may need to be further verified and studied in other databases. However, at this stage, we still believe that the nine genes signature proposed in this research has the capacity to accurately predict the patients’ prognosis with melanoma and provide a new direction for new treatment strategies.

## Conclusions

In conclusion, our research shows that the ferroptosis genes expression is related with the progression of patients of melanoma. We combined the ferroptosis gene expression of patients with clinical data to construct a signature containing 9 genes to accurately forecast the melanoma patients’ prognosis of. The research also revealed the close relationship between immune function and ferroptosis related genes through analysis of immune cell infiltration and immune-related functions.

## Supplementary Information

**Additional file 1.**

**Additional file 2.**

## Data Availability

The datasets generated and analysed during the current study are available in the TCGA repository. https://tcga-data.nci.nih.gov/tcga. The independent validation cohort data is available in the GEO repository. https://www.ncbi.nlm.nih.gov/geo( GSE65904 and GSE22153).

## Abbreviations

FRGs: Ferroptosis-related genes
TCGA: The cancer genome atlas
LASSO: Least absolute shrinkage and selection operator
OS: Overall survival
GPX4: Glutathione peroxidase 4
PCA: Principal component analysis
t-SNE: T-distributed stochastic neighbor embedding
ROC: Receiver operating characteristic curve
GO: Gene ontology
KEGG: Kyoto encyclopedia of genes and genomes
FDR: False discovery rate
TME: Tumor microenvironment
ssGSEA: Single-sample geneset enrichment analysis
qRT-PCR: Quantitative reverse transcriptase polymerase chain reaction
DEGs: Differential expression genes
DCs: Dendritic Cells; ACSL4:acyl-CoA synthetase long-chain family member 4
ALOX5: 5-Lipoxygenase
MT: Metallothionein
ZEB1: Zinc finger E-box-binding homeobox 1
ATP5MC3: ATP synthase membrane subunit c locus 3
CHAC1: ChaC glutathione specific gamma-glutamylcyclotransferase 1
CS: Citrate synthase
ACACA: Acetyl-CoA carboxylase alpha
ABCC1: ATP binding cassette subfamily C member 1
MTs: Metallothioneins
ABC: ATP binding cassette
MRP1: Multidrug resistance protein 1
GPX4: Glutathione peroxidase4
