# Supplementary material for: Identification and validation of a prognostic model for melanoma patients with 9 ferroptosis-related gene signature
Source: BMC Genomics. 2022 Mar 30;23:245. doi: 10.1186/s12864-022-08475-y (PMC8969311; doi:10.1186/s12864-022-08475-y)
Supplement: Supplementary file 1 — Additional file 1. [file 12864_2022_8475_MOESM1_ESM.docx]

**Table S1: Clinical characteristic of the melanoma patient used in this study**

|  | training cohort | validation cohort |
| --- | --- | --- |
| No. of patients | 318 | 137 |
| Age (%) |  |  |
| ≤65 | 208(65.4) | 89(65) |
| ＞65 | 110(34.6) | 48(35) |
| Gender (%) |  |  |
| Female | 117(36.8) | 56(40.9) |
| Male | 201(63.2) | 81(59.1) |
| Stage (%) |  |  |
| I | 67(21.1) | 26(19.1) |
| II | 98(30.8) | 38(27.7) |
| III | 115(36.2) | 55(40.1) |
| IV  unknow | 12(3.8)  26(8.1) | 10(7.3)  8(5.8) |
| Survival status |  |  |
| OS day (median) | 1186.5 | 1032 |
| Ending (%) |  |  |
| Survival | 176(55.3) | 61(55.5) |
| Death | 143(44.7) | 76(44.5) |

**Table S2: 60 ferroptosis-related genes**

ACSL4

AKR1C1

AKR1C2

AKR1C3

ALOX15

ALOX5

ALOX12

ATP5MC3

CARS1

CBS

CD44

CHAC1

CISD1

CS

DPP4

FANCD2

GCLC

GCLM

GLS2

GPX4

GSS

HMGCR

HSPB1

CRYAB

LPCAT3

MT1G

NCOA4

PTGS2

RPL8

SAT1

SLC7A11

FDFT1

TFRC

TP53

EMC2

AIFM2

PHKG2

HSBP1

ACO1

FTH1

STEAP3

NFS1

ACSL3

ACACA

PEBP1

ZEB1

SQLE

FADS2

NFE2L2

KEAP1

NQO1

NOX1

ABCC1

SLC1A5

GOT1

G6PD

PGD

IREB2

HMOX1

ACSF2

**Table S3: 16 immune-related cells and 13 immune-related pathways**

aDCs

APC_co_inhibition

APC_co_stimulation

B_cells

CCR

CD8+_T_cells

Check-point

Cytolytic_activity

DCs

HLA

iDCs

Inflammation-promoting

Macrophages

Mast_cells

MHC_class_I

Neutrophils

NK_cells

Parainflammation

pDCs

T_cell_co-inhibition

T_cell_co-stimulation

T_helper_cells

Tfh

Th1_cells

Th2_cells

TIL

Treg

Type_I_IFN_Reponse

Type_II_IFN_Reponse

**Table S4：The primers used in this study**

|  | forward primer (5'→3') | reverse primer (5→3') |
| --- | --- | --- |
| ACSL4 | ACTGGCCGACCTAAGGGAG | GCCAAAGGCAAGTAGCCAATA |
| ALOX5 | ATCAGGACGTTCACGGCCGAGG | CCAGGAACAGCTCGTTTTCCTG |
| ABCC1 | ATGTCACGTGGAATACCAGC | GAAGACTGAACTCCCTTCCT |

**Figure S1: The results of multivariate Cox regression OS analyses after adding ESTIMATE score.**


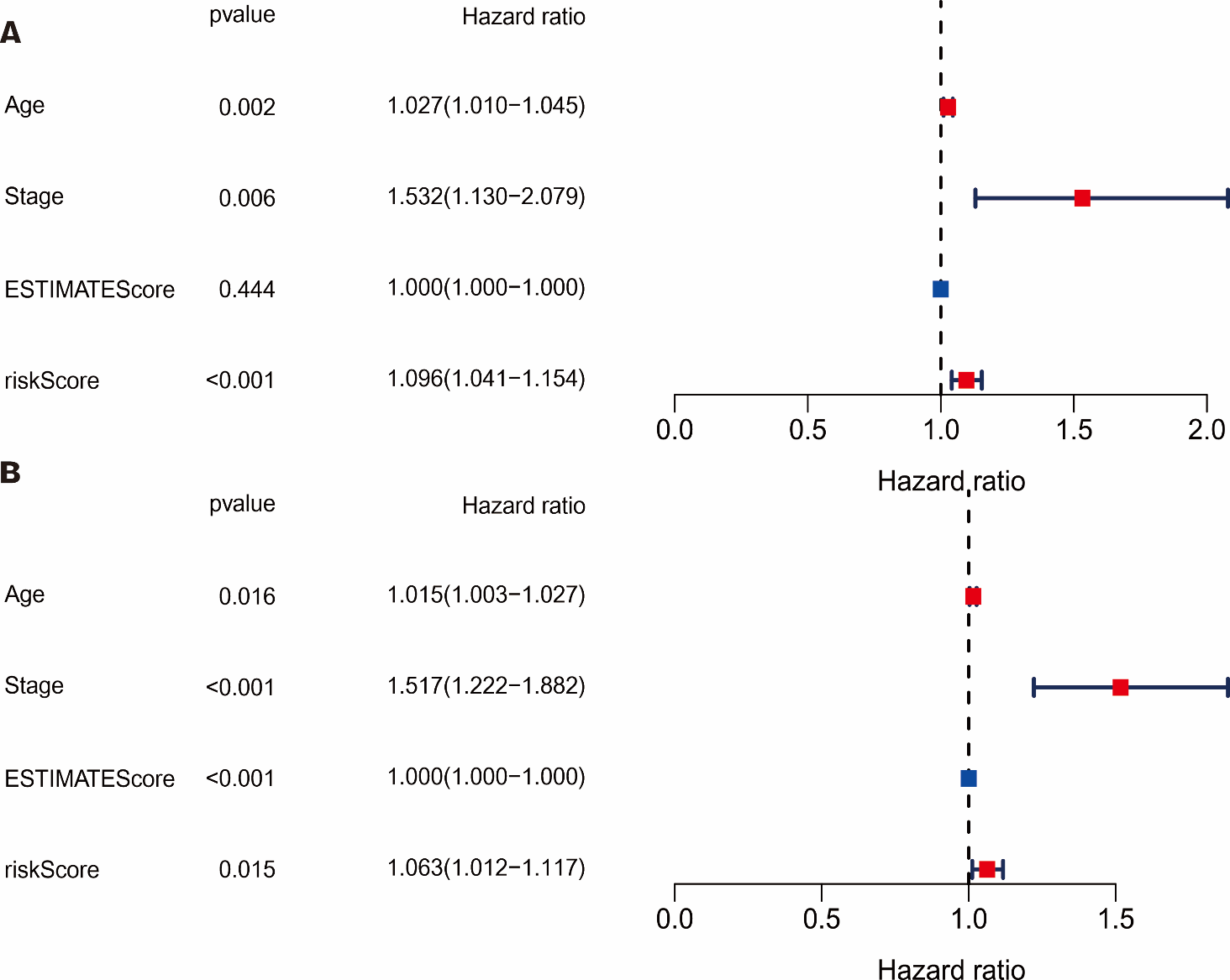
Figure S1. multivariate Cox regression OS analyses results in the validation cohort (A) and train cohort (B).
