# Supplementary figures and images for: Identification and validation of a prognostic model for melanoma patients with 9 ferroptosis-related gene signature
Source: BMC Genomics. 2022 Mar 30;23:245. doi: 10.1186/s12864-022-08475-y (PMC8969311; doi:10.1186/s12864-022-08475-y)

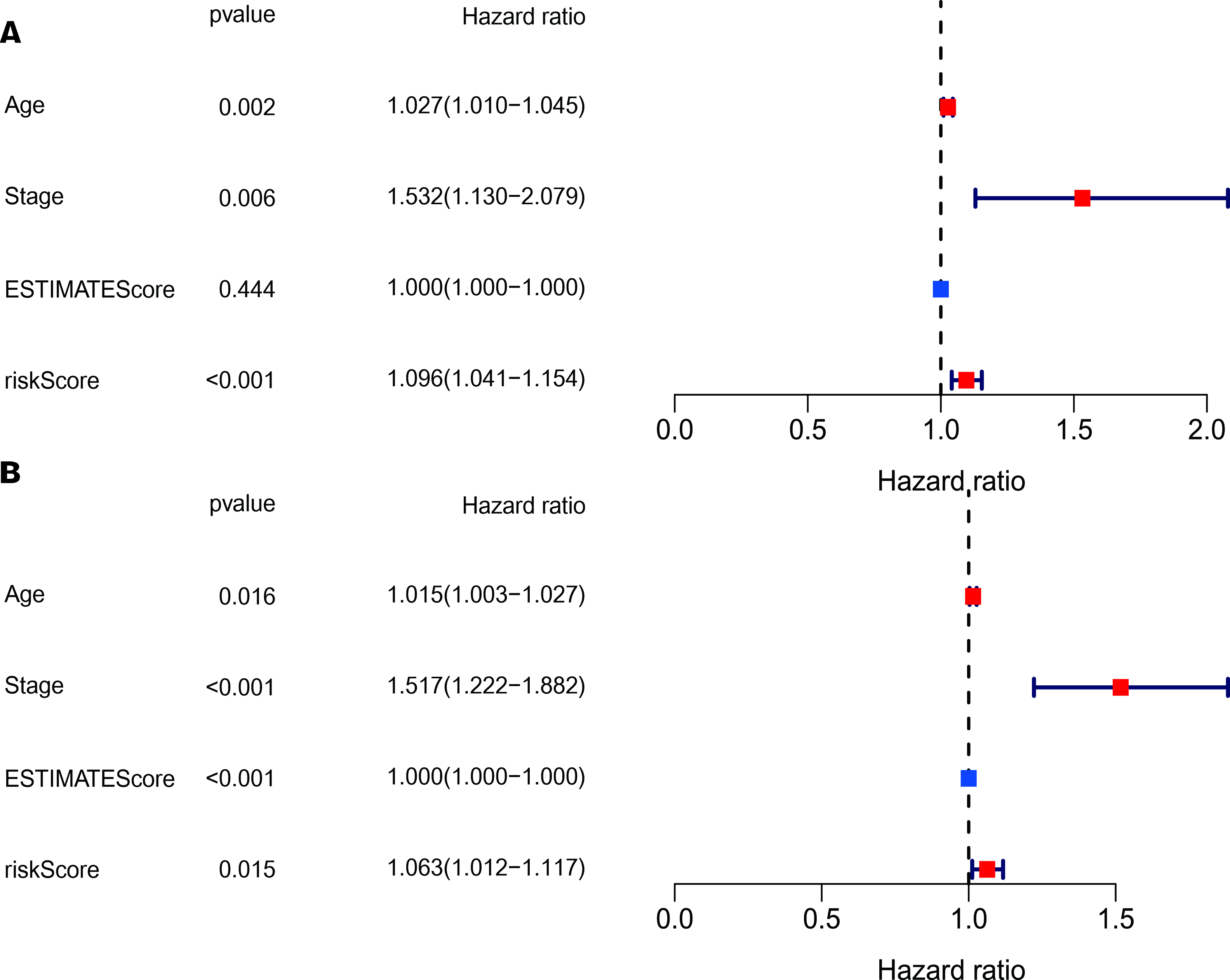

Supplement: Supplementary file 2 — Additional file 2. [file 12864_2022_8475_MOESM2_ESM.tif]
